# Evaluation de l’état vaccinal contre l’hépatite B et portage de l’Ag HBs chez le personnel médical et paramédical de l’Hôpital Central de Yaoundé, Cameroun

**DOI:** 10.11604/pamj.2013.16.111.2760

**Published:** 2013-11-23

**Authors:** Dominique Noah Noah, Guy Pascal Ngaba, Servais Fiacre Eloumou Bagnaka, Constant Assi, Emmanuel Ngantchet, Oudou Njoya

**Affiliations:** 1 Service de gastroentérologie de l’Hôpital Central de Yaoundé, Faculté de Médecine et des Sciences Pharmaceutiques de l’Université de Douala, Cameroun; 2 Hôpital de district de Bonassama, Faculté de Médecine et des Sciences Pharmaceutiques de l’Université de Douala, Cameroun; 3 Service de gastroentérologie de l’Hôpital général de Douala, Faculté de Médecine et des Sciences Pharmaceutiques de l’Université de Douala, Cameroun; 4 CHU de Cocody, UFR des Sciences Médicales, Abidjan, Côte d'Ivoire; 5 Service de gastroentérologie de l’Hôpital Central de Yaoundé, Cameroun; 6 Service de gastroentérologie du Centre hospialier Universitaire de Yaoundé, Faculté de Médecine et des SciencesBiomédicales de l’Université de Yaoundé 1, Cameroun

**Keywords:** Hépatite virale B, prévalence, personnel de santé, vaccination, Hôpital Central de Yaoundé, Viral hepatitis B, prevalence, health personnel, vaccination, Yaoundé Central Hospital

## Abstract

**Introduction:**

L’hépatite virale B est une affection à haut risque pour le personnel de santé. Au Cameroun, la vaccination contre le virus de l’hépatite B n'est pas obligatoire pour le personnel de santé. Le but de l’étude était d'évaluer l’état vaccinal contre l’hépatite virale B et la prévalence de l’Antigène HBs au sein du personnel médical et paramédical de l’Hôpital Central de Yaoundé.

**Méthodes:**

Il s'agissait d'une étude prospective, transversale menée à l’Hôpital Central de Yaoundé du 1er juillet au 31 octobre 2011. Toute personne membre du corps médical et paramédical ayant signée la fiche de consentement éclairé était incluse dans l’étude. Ceux ayant refusé de signer la fiche de consentement éclairé ou dont la fiche d'enquête n'avait pas été complètement remplie étaient exclus de l’étude. Une recherche systématique de l’antigène HBs était effectuée chez chaque personne incluse dans l’étude.

**Résultats:**

Au total 282 personnes ont participé à l’étude sur 760, soit un taux de participation de 37,1%. Les personnes ne connaissant pas leur statut sérologique vis-à-vis du virus de l’hépatite B était au nombre de 221 (85%). Deux cent vingt-cinq (86,5%) personnes n'étaient pas vaccinées contre l’hépatite B. Les personnes ayant reçu moins de 3 doses du vaccin contre l’hépatite virale B étaient au nombre de 23 (8,8%). Douze personnes (4,6%) déclaraient avoir reçu les 3 doses du vaccin contre l’hépatite virale B. La prévalence de l’antigène HBs était de 6,6% (n = 12).

**Conclusion:**

Cette étude montre que la prévalence de l’hépatite virale B reste élevée chez le personnel de l’Hôpital Central de Yaoundé (6,6%). Il ya un besoin de sensibilisation du personnel médical aux risques liés à l’exercice de leur profession.

## Introduction

L’hépatite virale B (HVB) est un problème de santé publique en Afrique [1]. L′Organisation Mondiale de la Santé (OMS) estime à deux milliards le nombre de personnes infectées par le virus de l’HVB parmi lesquels 350 millions sont des porteurs chroniques du virus [2, 3]. L’HVB est le principal facteur de risque du carcinome hépatocellulaire en Afrique [4].

Le personnel de santé est 4 fois plus à risque de contamination du virus de l’HVB que la population générale [5]. On estime que dans les hôpitaux 30 accidents d'exposition au sang surviennent pour 100 lits par an [6]. Des études menées au Cameroun chez les donneurs de sang et les pygmées ont retrouvé une prévalence de l’Ag HBs de 10,7% et 11,8% respectivement [7, 8]. On dispose depuis 1982 d′un vaccin contre l′hépatite B dont l′efficacité est prouvée par plusieurs études [9–11]. La réunion internationale de consensus contre le VHB qui s'est tenue à Paris en 2003 a recommandé de vacciner les personnes ayant un risque élevé d'exposition à ce virus, en particulier les professionnels de santé et les sujets exposés en raison d'une situation ou d'un comportement à risque [12].

Au Cameroun, la vaccination anti-VHB n′est pas obligatoire pour le personnel de santé ni de manière systématique ni en prophylaxie post-exposition. En 2006, une étude faite chez les personnels de santé à Enugu au Nigéria a montré que respectivement 22,4% et 3,7% des personnes étaient vaccinées contre l’HVB et avaient reçu les trois doses du vaccin anti-VHB malgré le risque potentiel lié à leur profession [13]. Au Cameroun, peu d'étude ont été publié sur l’épidémiologie de l’HVB chez le personnel de santé.

Le but de notre étude était d′évaluer l’état vaccinal contre l’HVB et la prévalence de l’antigène HBs (AgHBs) chez le personnel médical et paramédical de l′Hôpital Central de Yaoundé.

## Méthodes

Il s′agissait d′une étude prospective, transversale réalisée à l’aide d'un questionnaire auto-administré et d'un dosage sanguin systématique de l’antigène HBs. L’étude a été réalisée à l’Hôpital Central de Yaoundé (HCY) et au laboratoire d'analyses biomédicales de l’Hôpital de district de Bonassama du 1er juillet au 31 octobre 2011. La population d′étude était constituée de l’ensemble du personnel médical et paramédical de l′HCY.

Tout membre du personnel médical ou paramédical de l’hôpital était inclus dans l’étude après signature d'un consentement éclairé. Parmi eux, ceux refusant de signer le consentement écrit et ceux dont la fiche d'enquête était incomplètement renseignée étaient exclus. Le recrutement a été consécutif non probabiliste et volontaire après une sensibilisation menée dans tous les services. La fiche d'enquête comprenait des paramètres socio-démographiques (identité, sexe, âge, profession), les antécédents (transfusion, ictère, toxicomanie intraveineuse, scarification, tatouage, comportement sexuel à risque tel que la multiplicité du nombre de partenaires sexuels, l’utilisation des préservatifs, consommation d'alcool > 30 grammes par semaine), la connaissance sur l’HVB et le nombre de dose de vaccin contre l’HVB reçu (0,1,2 ou 3). Une personne était considérée correctement vaccinée si elle déclarait avoir reçu (sans contrôle du carnet de vaccinal ni du dosage de l’anticorps anti VHB) les trois doses du vaccin anti-VHB.

Un planning a été établi précisant l′ordre de passage par service. Chaque membre du personnel participant à l’étude remplissait au laboratoire une fiche d'enquête numérotée. Ce numéro était porté sur le tube de prélèvement sanguin dans un but d′anonymat. Les prélèvements étaient effectués par le personnel du laboratoire de l′Hôpital Central Yaoundé dans des conditions d'asepsie et de sécurité adéquates. Les échantillons de sang étaient recueillis dans des tubes secs stériles préalablement étiquetés puis laissés sédimentés ou centrifugés, aliquotés et congelés. Les échantillons étaient transportés le même jour à Douala dans une glacière au laboratoire de l′Hôpital de District de Bonassama pour analyse. La liste nominative était retenue pour préserver l’anonymat.

Un test rapide immunochromatographique (One Step HBsAg Test Disk) était utilisé pour le dépistage et les échantillons positifs étaient confirmés par un test immunoenzymatique ELISA (PATHZYME^®^ HBsAg).

### Aspects éthiques

Les résultats sanguins obtenus étaient transmis aux participants par le gastroentérologue de l′Hôpital Central de Yaoundé suivant un ordre qui leur était communiqué. Les personnes ayant un test positif étaient prises en charge à l′Hôpital Central de Yaoundé par un gastroentérologue. Les personnes ayant un résultat négatif étaient proposées à la vaccination contre le virus de l′hépatite B. Une clairance éthique a été obtenue auprès du Comité National d'Ethique du Cameroun. Tous les participants à l’étude avaient signé un consentement éclairé écrit.

### Analyse statistique

L’analyse statistique a été réalisée en utilisant le logiciel R software version 2.12.1 en excluant tous les participant possédants les données manquantes. La saisie des données a été faite en utilisant le logiciel Microsoft, Excel version 2007. Pour la comparaison de deux variables quantitatives nous avons utilisé le test de khi2 lorsque chaque case de la table de contingence avait une valeur au moins égale à 5. Lorsqu'au moins une case des tables de contingence avait une valeur inférieure à 5 le test de Fisher était utilisé. Le P value des tests était comparé au seuil de 5%.

## Résultats

Au total 282 personnes ont participé à l’étude sur les 760 membres du personnel médical et paramédical que compte l’HCY, soit un taux de participation de 37,1%. Parmi ces personnes 260 remplissaient les critères d'inclusion soit 92,1%. Le sexe ratio était de 0,43 (78 hommes contre 182 femmes).

Les personnes ayant participé à notre étude étaient âgés de 21 à 57 ans. La tranche d'âge la plus représentée était celle de 41-50 ans (34,3%), suivie de celle de 31-40 ans (28,7%). La moyenne d'âge de notre échantillon était de 40,69 ans ± 9,29 ans.

Les infirmiers étaient majoritaires dans notre échantillon 187 (71,92%). Le nombre de personnes mariées étaient de 153 (58,84%), 90 (34,61%) étaient célibataires, 8 (3, 01%) étaient veufs et 5 (1,92%) étaient divorcés. Les personnels ayant participé à l’étude ne connaissant pas leur statut sérologique HVB était au nombre de 221 (85%). 225 (86,5%) personnels n'étaient pas vacciné contre l’hépatite B (Tableau 1). Respectivement 8,8% (n = 23) et 4,6% (n = 12) des personnes étaient insuffisamment et correctement vaccinées (Tableau 2).

**Tableau 1:** Répartition de la population d'étude vaccinée et non vaccinée en fonction de la profession et des tranches d'âge

	Etat vaccinal			
Statut vaccinal	Non vaccinés (%)	Vaccinés (%)	Population totale	P-value
	225 (86.5)	35 (13.5)	260	
**Profession**	**225 (86.5)**	**35 (13.5)**	**260 (100.0)**	0.17
auxiliaire de santé	48 (18.5)	4 (1.5)	52 (20.0)	
Infirmier	161 (61.9)	26 (10.0)	187 (71.9)	
Médecin	16 (6.1)	5 (2.0)	21 (8.1)	
**Tranche d'âge**	**225 (86.5)**	**35 (13.5)**	**260 (100.0)**	0.78
21-30	38 (14.6)	4 (1.5)	42 (16.1)	
31-40	66 (25.4)	9 (3.5)	75 (28.9)	
41-50	75 (28.8)	14 (5.4)	89 (34.2)	
51-60	46 (17.7)	8 (3.1)	54 (20.8)	

**Tableau 2:** Répartition de la population d'étude suivant le statut sérologique AgHBs

	Sérologie AgHBS			
Statut sérologique	Négatif (%)	Positif (%)	Total (%)	p. value
**Profession**	**243 (93.5)**	**17 (6.5)**	**260 (100)**	**0.02**
Infirmier	178 (68.5)	9 (3.5)	187 (71.9)	
Auxiliaire de santé	44 (16.9)	8 (3.1)	52 (20.0)	
Médecin	21 (8.1)	0 (0)	21 (8.1)	

Les personnes porteuses de l’Ag HBs étaient au nombre de 17 (6,6%) dont aucun médecin (P < 0.05) (Tableau 3). Des 17 porteurs de l’AgHBs, 8 (3,1%) étaient de sexe masculin (P < 0.05).

**Tableau 3:** Répartition de la population d'étude suivant le nombre de doses de vaccin reçu

Nombre de dose	Nombre de personnes	Fréquence(%)
0	225	86.5
1	16	6.2
2	7	2.7
≥3	12	4.6
**Total**	**260**	**100**

## Discussion

Au total 282 personnes de l’HCY ont participé à notre étude sur les 760 personnes qui y travaillent, soit un taux de participation de 37,10%. Ce faible taux de participation à notre étude peut avoir plusieurs explications. Notre étude avait un caractère universitaire (non officiel donc sans contrainte morale) d'une part, et la problématique des hépatites chroniques dans notre contexte sort du cadre hospitalier. En effet, la majorité de la population (y compris le personnel soignant) considère que les hépatites virales ne se traitent pas à l’hôpital. D'autre part, la notion de la prise de sang est suspecte dans la mesure où certaines personnes pensaient que c'était un moyen détourné pour faire le dépistage du VIH/SIDA. Ces résultats contrastent avec ceux de Fiona Braka et al. dans une enquête réalisée en Ouganda en 2006 qui ont trouvé un taux de participation de 93,4% chez les personnels de santé [14].

Selon le sexe la population de notre étude est majoritairement féminine (69%). Ceci peut s'expliquer par le fait de la forte proportion des femmes dans la profession infirmière. Ces résultats sont en rapport avec ceux de Fiona Braka qui a trouvé une proportion du personnel féminin de 57% en Ouganda [14].

L’âge des sujets variait entre 21 et 57 ans avec une moyenne d'âge de 40,69 ± 9.29 ans. Ces résultats se rapprochent de ceux obtenus par Djeriri et al. qui ont obtenu 41,4 ± 7ans [15]. Des résultats similaires ont été observés par Fiona Braka et al. [14]. La moyenne d'âge très élevée dans notre étude peut s'expliquer du fait de la profession infirmière et paramédicale qui constitue la majeure partie de notre échantillon est vieillissante au Cameroun. En effet, depuis plus de 20 ans il y a eu un arrêt de recrutement du personnel infirmier et paramédical dans la fonction publique camerounaise au détriment des médecins qui sortent des facultés de médecine d'Etat et qui sont directement recrutés dans la fonction publique.

Concernant les connaissances relatives à l’HVB, 85% des personnels ayant participé à l’étude ignorait leur statut sérologique. Concernant la vaccination contre l’HVB, 86,5% n'étaient pas vacciné. Les personnels partiellement vaccinés étaient au nombre de 23 (8,8%). Dans une étude menée en Ouganda, Fiona Braka et al. ont montré que seulement 5,1% des personnels de santé avaient reçu au moins une dose de vaccin et 98,1% étaient prêt à le recevoir si recommandé après les tests de laboratoire [14]. Dans un autre régistre, Djeriri K. et al. Dans une étude menée au Maroc ont montré que 98% de leur population d'étude pensaient que la vaccination était nécessaire dans la prévention contre l’HVB [15].

Les personnels correctement vaccinés étaient 12 (4.6%). Ces résultats montrent le peu d'intérêt et surtout le degré d'ignorance qu'accuse notre population d'étude, ceci étant d'autant plus grave qu'il s'agit d'un personnel de santé. Une autre explication peut venir du fait qu'il n'ya pas de politique sanitaire basée sur la formation du personnel de santé sur les risques professionnels en général et sur ceux liés au VHB en particulier. Il ya là une nécessité non seulement d'inclure la sensibilisation dans la pratique courante, d'équiper les services de soins en équipement adéquat (dispositifs de lavage des mains, équipements appropriés, stérilisation du matériel d'usage en adéquation avec les normes internationales…), mais aussi d'inclure dans la formation initiale la sensibilisation en vue de la prévention des risques professionnels en général et ceux liés à l’HVB en particulier.

Concernant les risques liés à la profession médicale, EPINET et al. [6] ont montré dans une étude publiée dans Health Devices en 1999 que 30 accidents d'exposition au sang surviennent au Brésil par 100 lits et par an. Par ailleurs, le risque de contacter l’HVB par le personnel de santé est quatre fois plus élevé que dans la population générale [5]. D'autre part, le traitement de l’HVB très avancé actuellement dans les pays de l’occident et qui associe les antiviraux et les immuno modulateurs n'est toujours pas accessible dans nos pays. C'est pourquoi l’OMS recommande la vaccination contre l’HVB comme le seul moyen efficace de lutte contre cette maladie associé aux mesures d'hygiène hospitalière [2].

Au Cameroun, il n'y a pas de programme national de lutte contre les hépatites virales alors que ces programmes existent dans les pays à faible endémicité où le risque est moins important. Dans les pays à basse prévalence HBV (< 2%) comme l’Amérique du nord, l’Europe de l’ouest, des programmes de lutte contre l’HVB ont été initiés dès les années 1980. Dans les pays d'endémicité similaire (> 8%) comme la Cote d'Ivoire et le Sénégal il existe des programmes de lutte contre les hépatites virales. En France, cette stratégie associée au renforcement des précautions universelles ont donné comme résultats la diminution du portage de l’Ag HBs chez des personnes à risque telles que les personnels de santé [12]. D'après la déclaration de Rabat [4], l’HVB pourrait être éliminé par la mise en place d'une vaccination universelle, avec comme avantage d'éliminer la principale cause du carcinome hépatocellulaire (CHC). Le succès des programmes de vaccination est documenté dans les pays à forte endémie tels que Taïwan, Gambie où la prévalence du VHB est passée de 10 à 1,1 et 0,6% respectivement après introduction des programmes de vaccination [4].

Concernant les personnels vaccinés (13,5%), la plupart l’avait été dans les campagnes de vaccination sans dépistage préalable. Malheureusement dans notre étude nous n'avons pas pu tester le taux de protection anti VHB pour déterminer l′efficacité de cette vaccination.

La prévalence de l’AgHBs était de 6.6% dans notre population d'étude. Cette prévalence est inférieure à celle des groupes des donneurs de sang (10,7%) et des pygmées (11%) retrouvée dans deux études réalisées respectivement à l’HCY et au sud du Cameroun [7, 8]. On note également des taux élevés de prévalence chez les personnels de santé en Ouganda et au Burkina Faso [13, 15].

Ce taux est nettement supérieur à ceux observés par plusieurs études réalisées au Maroc et au Brésil [15–17]. Ceci peut s'expliquer par le niveau socioéconomique plus élevé de la population dans ces pays et aussi par le fait que le Maroc et le Brésil sont situés dans la zone d'endémicité intermédiaire.

Parmi le personnel positif pour l’Ag HBs, on notait uniquement les infirmiers et paramédicaux et pas de médecin (P = 0.02). Ces résultats se rapprochent de ceux retrouvés en Afrique de l’ouest [13, 15]. La présence de l’Ag HBs uniquement chez les infirmiers et paramédicaux peut s'expliquer par leur nombre important. En effet dans notre échantillon on avait seulement 21 médecins soit 8,08% de l’échantillon. Une autre explication peut provenir du fait que les infirmiers sont ceux qui sont le plus en contact étroit avec les malades et qui leur font les soins avec tous les risques connus.

La tranche d'âge la plus touchée était celle de 31 à 40 ans avec un pourcentage de 43.8% suivi de celle de 41 à 50 ans (25%). Cette différence n'est pas statistiquement significative (P = 0.52). Par contre, Braka et al. [14] a retrouvé une augmentation de la prévalence de l’AgHBs avec l’âge.

## Conclusion

Cette étude montre que la prévalence de l’hépatite virale B reste élevée à l’Hôpital Central de Yaoundé (6,6%). Il y a un besoin de sensibilisation du personnel médical aux risques liés à l’exercice de leur profession
